# The functional role of electrophysiological heterogeneity in the rabbit ventricle during rapid pacing and arrhythmias

**DOI:** 10.1152/ajpheart.00894.2012

**Published:** 2013-02-22

**Authors:** Martin J. Bishop, Edward J. Vigmond, Gernot Plank

**Affiliations:** ^1^Biomedical Engineering Department, Division of Imaging Sciences, King's College London, London, United Kingdom;; ^2^Institut LIRYC, Université Bordeaux 1, Bordeaux, France; and; ^3^Institute of Biophysics, Medical University of Graz, Graz, Austria and Oxford e-Research Centre, University of Oxford, Oxford, United Kingdom

**Keywords:** electrophysiological heterogeneity, computational modeling, arrhythmias

## Abstract

Electrophysiological heterogeneity in action potential recordings from healthy intact hearts remains highly variable and, where present, is almost entirely abolished at fast pacing rates. Consequently, the functional importance of intrinsic action potential duration (APD) heterogeneity in healthy ventricles, and particularly its role during rapidly activating reentrant arrhythmias, remain poorly understood. By incorporating both transmural and apicobasal APD heterogeneity within a biventricular rabbit computational model and comparing with an equivalent homogeneous model, we directly investigated the functional importance of intrinsic APD heterogeneity under fast pacing and arrhythmogenic protocols. Although differences in APD were significantly modulated at the tissue level during pacing and further reduced as pacing frequency increased, small differences were still noticeable. Such differences were further marginally accentuated/attenuated via electrotonic effects relative to wavefront propagation directions. The remaining small levels of APD heterogeneity under the fastest pacing frequencies resulted in arrhythmia initiation via heterogeneous conduction block, in contrast to complete block in the homogeneous model. Such induction mechanisms were more evident during premature stimuli at slower paced rhythms where intrinsic heterogeneity remained to a greater degree. During sustained arrhythmias, however, intrinsic heterogeneity made little difference to overall reentrant behavior, either visually, or in terms of duration, metrics quantifying filament/phase singularity dynamics, and global electrocardiogram characteristics. These findings suggest that, despite being important during arrhythmia initiation, intrinsic electrophysiological heterogeneity plays little functional role during rapid pacing and sustained arrhythmia dynamics in the healthy ventricle and thus questions the need to incorporate such detail in computational models when simulating rapid arrhythmias.

electrophysiological heterogeneity in cellular ionic currents exists in the ventricular myocardium of many species (2). In cells isolated from different spatial locations within the ventricles, these differences in electrical membrane dynamics manifest themselves as distinct differences in action potential durations (APDs) and morphologies. For example, the APD of isolated endocardial cells is significantly longer than that of isolated epicardial cells (35, 36, 52), with epicardial cells also showing a distinctive repolarization (or “notch”) immediately after the upstroke (35). Ventricular cells with distinct electrophysiological characteristics, termed M cells (2, 3), have also been identified, primarily in the midmyocardium, of many species, including rabbit (36) and humans (22). In single cell recordings, M cells display APDs that are much longer than epi- or endocardial cells, particularly at slow pacing rates. Furthermore, in addition to transmural heterogeneity, APD heterogeneity has also been highlighted between the apical and basal regions (10, 26).

Despite the significant electrophysiological heterogeneity at the single cell level, how these differences are expressed in well-coupled ventricular tissue, both in vitro and in vivo, remains somewhat controversial (3, 44, 50). Recordings from intact perfused ventricular wedge preparations have suggested that, although heterogeneity is present at the tissue level (22, 53), the magnitude of the transmural dispersion of APD is significantly modulated by electrotonic interactions (3, 42) and attenuated even further during rapid pacing rates (22, 53). Furthermore, in vivo recordings from intact hearts (44), in addition to more recent tissue level recordings (38), have not conclusively defined the intrinsic APD heterogeneity in healthy ventricles (44). Although methodological considerations regarding experimental preparations and clinical procedures have been suggested to underlie such disparities (3, 44), this is in stark contrast to recordings during pathological conditions, such as inherited ion channelopathies and heart failure. Here, dispersion of repolarization due to APD heterogeneity has not only been well characterized but directly related to increased arrhythmogenic risk (1). Therefore, in the intact heart during physiological conditions, the functional presence and importance of electrophysiological heterogeneity, and particularly its association with an arrhythmogenic substrate, remain unclear.

Incorporating intrinsic electrophysiological ionic heterogeneity may therefore represent an important consideration in the construction and use of computational ventricular models aimed at the study of arrhythmia mechanisms (6). Indeed, the vast majority of the models used for such investigations (13, 47, 51), including many from our own group (5, 6), represent the ventricles as homogeneous. In light of these shortcomings, it is often argued that the inclusion of ionic heterogeneity would not be expected to significantly alter the findings of the studies related to arrhythmia mechanisms, often citing the modulation of APD heterogeneity due to electrotonic coupling at the tissue level (3, 42) and at fast rates associated with reentrant activity (22, 53). The close agreement of the simulated reentrant dynamics in previous computational studies using homogeneous models with corresponding experimental (5, 13, 45) and clinical measurements (47) suggests that this is the case. However, previous simulation studies on simplified regular slab geometries have indicated that assigned transmural heterogeneity in APD can destabilize reentrant activity in an important manner (14, 15). A thorough assessment of the interaction of electrophysiological heterogeneity with reentrant dynamics within the complex biventricular anatomy is so far lacking. Such a study will allow us to gauge when we need to incorporate heterogeneity within computational models.

In this study, we incorporate both transmural and apicobasal intrinsic electrophysiological heterogeneities within a rabbit whole ventricular computational model. Under different rapid pacing protocols, we assess the electrotonic modulation of corresponding intrinsic gradients in APD and their interaction with global wavefront direction with respect to ventricular anatomy. Through comparison with an electrophysiologically homogeneous model, we conduct a thorough qualitative and quantitative assessment of the importance of intrinsic electrophysiological APD heterogeneity in the initiation of arrhythmias via pacing and in the reentrant dynamics of sustained arrhythmias within the healthy rabbit ventricles.

## METHODS

### Computational Model

Electrical activity was simulated within a tetrahedral finite element model of the rabbit ventricles, previously described (17). The model represents gross biventricular geometry and includes anatomically realistic fiber architecture but lacks detail regarding fine scale anatomical features such as the coronary vasculature and endocardial structures. The model contains 547,680 myocardial nodes defining 2,966,136 tetrahedral elements. The mean finite element edge length within the myocardium is 279 μm. In addition to the ventricular myocardium, the model also represents a perfusing conducting bath surrounding the ventricles and within ventricular cavities. Two versions of the model were used in the study: a heterogeneous model that included transmural and apicobasal electrophysiological heterogeneity (see below), and a homogeneous model that assigned the same electrophysiological parameters throughout.

### Simulating Electrical Activity

#### Governing equations.

The bidomain equations were used to simulate electrical activation within the ventricular models (24)

(1) $$\nabla \cdot \sigma_{\text{i}}\nabla \phi_{\text{i}}=\beta I_{\text{m}}$$

(2) $$\nabla \cdot \sigma_{\text{e}}\nabla \phi_{\text{e}}=-\beta I_{\text{m}}-I_{\text{ei}}$$

(3) $$I_{\text{m}}=C_{\text{m}}\frac{\partial V_{\text{m}}}{\partial t}+I_{\text{ion}}\left(V_{\text{m}},\eta \right)-I_{\text{s}}$$

(4) $$\nabla \cdot \sigma_{\text{b}}\nabla \phi_{\text{e}}=-I_{\text{eb}}$$

where ϕ_i_ and ϕ_e_ are the intracellular and extracellular potentials, respectively; *V*_m_ = ϕ_i_ − ϕ_e_ is the transmembrane voltage; **σ**_**i**_ and **σ**_**e**_ are the intracellular and extracellular conductivity tensors, respectively, β is the membrane surface to volume ratio; *I*_m_ is the transmembrane current density; *I*_ei_ and *I*_eb_ are extracellular stimuli applied in the interstitial space or the bath, respectively; *I*_s_ is a transmembrane stimulus; *C*_m_ is the membrane capacitance per unit area,; and *I*_ion_ is the membrane ionic current density that depends on *V*_m_ and a set of state variables, **η**. The value of *C*_m_ was 1 μF/cm^−2^. At tissue boundaries, no flux boundary conditions are imposed for ϕ_i_ in the intracellular space whereas continuity of the normal component of the extracellular current (

$$\sigma_{\text{e}}\nabla \phi_{\text{e}}\cdot \text{n}$$)

and continuity of ϕ_e_ in the interstitial space are enforced along the tissue-bath interface. At the boundaries of the conductive bath surrounding the tissue, no flux boundary conditions for ϕ_e_ are imposed.

During certain protocols, it was preferable to represent the tissue as a single conducting domain, whereby the bidomain equations are reduced to the monodomain equation

(5) $$C_{\text{m}}\frac{\partial V_{\text{m}}}{\partial t}+I_{\text{ion}}=\nabla \cdot \sigma_{\text{m}}\nabla V_{\text{m}},$$

where **σ**_**m**_ is the harmonic mean conductivity tensor chosen to match bidomain conduction velocities (7). During monodomain simulations, no flux boundary conditions are imposed on *V*_m_. A monodomain representation was used for restitution and rapid pacing arrhythmia induction protocols, as well as simulating the long durations of sustained arrhythmia activity. Bidomain representations were used during external shock application for induction via shock-induced arrhythmogenesis (see below). Bidomain was used during stimulation to correctly capture formation of virtual electrodes. During pacing and sustained arrhythmias, bidomain effects play a very minor role, and so here the more computationally efficient monodomain approximation was used. Solutions to the bidomain and monodomain equations above were performed using the finite element method within the Cardiac Arrhythmia Research Package (CARP; Ref. 48), the underlying numerical details of which have been described extensively elsewhere (40, 49).

#### Electrophysiological parameters.

The membrane ionic current was represented by the recent rabbit ventricular cell model of Mahajan-Shiferaw (32). Modifications used to represent electrophysiological heterogeneity are described below. The Clerc (16) intra- and extracellular conductivities were used, being 0.17 S/m along the fiber and 0.019 S/m along the cross-fiber directions within the intracellular domain, and 0.62 S/m and 0.24 S/m, respectively, within the extracellular domain. The bath-loading effects on the morphology of the excitation wavefront are very minor when using these values and thus were neglected (7). During simulations of arrhythmias, conductivity values were uniformly scaled to reduce conduction velocity by 25% in all directions. Such a reduction is also commonly used in experimental studies through either the use of pharmacological uncoupling agents (31) or flecainide to slow conduction (29), due to difficulties in successfully sustaining reentrant activity in healthy rabbit ventricles (11, 31, 34). The bath conductivity, σ_b_, was set to 1.0 S/m (isotropic).

#### Electrophysiological heterogeneity assignment.

**Fig. 1.**
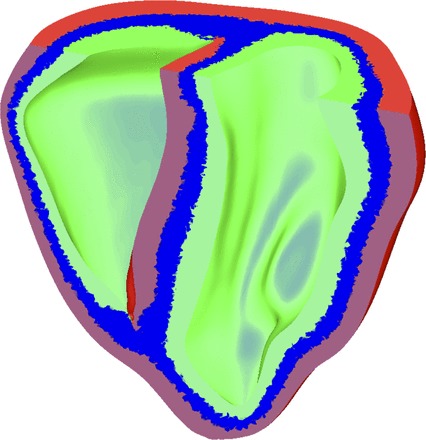
Assignment of transmural regions showing epicardial (red), midmyocardial (blue), and endocardial (green) layers.

Intrinsic electrophysiological heterogeneity was represented by modifying individual ionic currents based on both previous modeling studies (33, 43) and experimental data (23, 35, 36, 52). Specifically, maximum conductivities of the transient-outward current (*g*_tos_ and *g*_tof_), the sodium current (*g*_Na_), and the rapid (*g*_Kr_) and slow (*g*_Ks_) delayed-rectifier potassium currents within the Mahajan-Shiferaw model were varied to define distinct endocardial, midmyocardial, and epicardial regions. The spatial distribution of intrinsic transmural heterogeneity in ionic currents for these specific regions was assigned relative thicknesses of 3:3:2, respectively, across the myocardial wall (33, 43). The septum was defined as part of the left ventricle (LV) with the right ventricle (RV) side of the septum defined as “epicardial” tissue (26). Such a choice is in line with previous experimental studies (37), which have shown a decrease in APD across the septum (from LV to RV), suggesting a more epicardial-like electrophysiological make up on the RV side. The assignment of transmural regions within the ventricles is shown in Fig. 1. Table 1 lists the modifications made to the maximum conductances of the above-mentioned ionic currents to represent intrinsic transmural heterogeneity.

**Table 1.** Relative adjustments made to default values of maximum conductances of ionic currents within Mahajan-Shiferaw model

Current	Parameter	Endo	Mid	Epi
Transient outward (slow)	*g*_*tos*_	2.0	1.5	1.0
Transient outward (fast)	*g*_*tof*_	0.01	0.1	1.0
Fast sodium	*g*_*Na*_	0.8	0.9	0.7
Slow-inward potassium	*g*_*Ks*_	1.0	0.8	2.5
Delayed-rectifier potassium	*g*_*Kr*_	1.0	0.5	1.5

Endo, mid, and epi: endocardial, midmyocardial, and epicardial.

In addition to transmural heterogeneity, intrinsic apicobasal heterogeneity in repolarization was introduced via an alteration of the slow delayed rectifier potassium current *I*_Ks_. In the case of the rabbit, APD is longer at the apex than the base (10, 26). Therefore, an additional linear scaling function of *g*_Ks_ * (1.0 + *d*) as used previously (26) was applied, where *d* represents the normalized apex-base distance.

### Stimulation Protocol

#### Dynamic restitution.

**Fig. 2.**
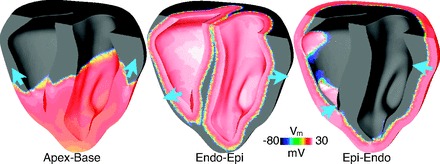
Transmembrane voltage (*V*_m_) distributions within the ventricles wavefront propagation directions following apical, endocardial, and epicardial stimulation.

Rapid pacing protocols were performed for three separate pacing directions: apex-base: pacing electrode located at the apex, inducing wavefront propagation in the apex-base direction; endo-epi: pacing electrode defined as the entire endocardial surface, inducing wavefront propagation in the endocardial-epicardial direction throughout the ventricles; and epi-endo: pacing electrode defined as the entire epicardial surface, inducing wavefront propagation in the epicardial-endocardial direction throughout the ventricles. Wavefront propagation directions for each protocol are shown in Fig. 2. Each protocol consisted of 10 paced beats at a particular basic cycle lengths (BCL), which was successively shortened from 500, 400, 300, 280, 260, 240, 220, 200, and 180 ms where 180 ms represented the shortest BCL at which 1:1 conduction was achieved. Although stimulation of the entire endocardial and epicardial surfaces does not represent experimental protocols, their use allowed induction of wavefront propagation approximately towards the epicardial/endocardial surfaces, respectively, throughout the ventricles. Furthermore, the endo-epi direction approximately represents activation via the Purkinje system. Throughout, restitution curves are plotted against BCL as opposed to diastolic interval (DI) to allow direct comparison in the response of the two models at different pacing rates and during different protocols. It should be noted that, during steady state, the point at which the gradient of the restitution curve of APD vs. DI has a value equal to 1 and the gradient of the restitution curve APD vs. BCL has a value of 0.5. More generally, the gradient of the APD vs. DI curve (*m*_DI_) can be related to the gradient of the APD vs. BCL curve (*m*_BCL_) via *m*_DI_ = *m*_BCL_/(1 − *m*_BCL_). In addition, restitution protocols were also performed at the single cell level.

#### Arrhythmia induction.

Susceptibility to arrhythmia induction was assessed using both rapid pacing and premature stimuli (S1S2) protocols with the pacing electrodes defined above. For the rapid pacing protocol, pacing was continued following the dynamic restitution protocols above, successively reducing the BCL in 5-ms increments from 180 to 160 ms. For the S1S2 protocol, premature stimuli of coupling interval (CI) between 215 and 160 ms were applied after the 10th beat following pacing at a constant cycle length of 400 ms.

The above pacing protocols did not reliably induce arrhythmias in both models. Therefore, to allow the dynamics of sustained reentrant arrhythmias to be analyzed in both models required the use of an established arrhythmogenic shock-induction protocol, as described previously (9), which reliably induced numerous episodes of arrhythmias in both models. Briefly, a monophasic truncated exponential shock (duration 5 ms) was delivered to the ventricles via a plate electrode setup, applied at a certain CI following the 10th preconditioning beat (apical stimulation, BCL 180 ms). Different combinations of shock strengths between 15 and 30 V and CIs between 160 and 240 ms were used to induce a total of 36 different arrhythmia episodes. Induced arrhythmia durations were defined as the time from shock-end until the entire ventricles returned to polarization levels of less than −80 mV.

### Data Analysis

#### APD calculation.

APDs were defined as the time point between depolarization (positive crossing of 0-mV threshold) and 90% repolarization and were calculated at all node points within the ventricles. Throughout, an average of APD of the eighth and ninth paced beat was used for analysis at each BCL.

#### ECG calculation.

Unipolar electrogram recordings were measured with an Einthoven configuration (8) and used to compute pseudo-electrocardiogram (ECG) traces. Dominant frequencies (DFs) were calculated directly from pseudo-ECG traces following preprocessing with a fast Fourier transform.

#### Filament analysis.

Filaments, which represent the organizing centers of reentrant circuits, were detected using an algorithm based on the approach of Fenton and Karma (19), adapted for use within an unstructured finite element regime (9). Briefly, the method defines the location of a filament as the intersection of the iso-surfaces of *V*_m_ = *V*_iso_ (where *V*_iso_ distinguishes polarized and depolarized regions of tissue) and

$$\frac{\text{d}V_{\text{m}}}{\text{d}t}=0$$.

The methods for identifying individual filaments in space and tracking their dynamics in time were based on previous studies (12, 47), adapted for use on unstructured finite element meshes used in this study, as detailed previously (9).

Throughout each simulation, all filament interactions (birth, death, division, and amalgamation) were recorded along with total filament numbers and mean lengths at each time step. Total filament interaction rate was defined as the sum of the birth, death, division and amalgamation rates. The intersections of filaments with epicardial triangles within the model were identified and counted as unique surface phase singularities (PSs). Maximum numbers of filaments and PSs were defined as the maximum total number of filaments/PSs present within the ventricles in any one time frame during a particular episode.

## RESULTS

### Electrotonic Modulation of Manifest Transmural APD Heterogeneity

**Fig. 3.**
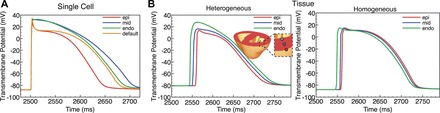
Transmural variation in assigned action potentials (APs). *A*: single cell APs for epicardial (red line), midmyocardial (blue line), and endocardial (green line) cells used in heterogeneous model, in addition to default AP (orange line) used in homogeneous model. *B*: APs recorded at tissue level from transmural locations shown in *inset* for the heterogeneous (*left*) and homogeneous (*right*) model during apical pacing at a basic cycle lengths (BCL) of 500 ms.

Figure 3*A* shows single cell AP traces incorporating the intrinsic changes in ionic currents associated with endo-, mid-, and epi-regions defined above, along with an AP defined by the default value of the Mahajan-Shiferaw model following pacing at a BCL of 500 ms. Here, *I*_Ks_ modifications are as a point mid-way between apex and base. APDs for single cells are 181 ms (endocardial), 199 ms (midmyocardial), 141 ms (epicardial), and 173 ms (default). Note that comparing additional epicardial APs located at the base and apex, APD dispersion is much less in the apicobasal direction with APDs of 135 and 148 ms, respectively.

These distinct differences seen in AP morphology at the single cell level are significantly modulated at the whole tissue level in the heterogeneous model due to electrotonic effects, as shown in Fig. 3*B*, *left*, which plots APs recorded from individual points within the endo-, mid-, and epi-regions during apical pacing at a BCL of 500 ms. For comparison, Fig. 3*B*, *right*, shows APs recorded from the same locations in the homogeneous model. APDs for the three respective locations in the heterogeneous model are 192 ms (endocardial), 188 ms (midmyocardial), and 162 ms (epicardial), compared with 173, 173, and 171 ms, respectively, in the homogeneous model. Note that in the heterogeneous model, electrotonic effects due to the close proximately of the epicardial layer of tissue to the midmyocardial tissue acts to reduce the APD of the midmyocardial tissue below that of the endocardial tissue.

### Restitution Analysis

#### Single cell.

**Fig. 4.**
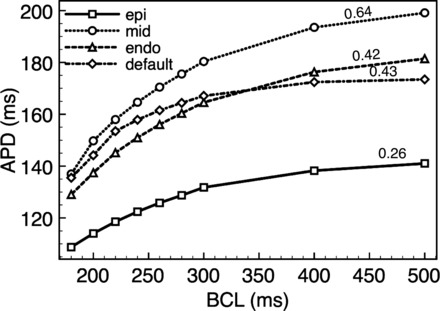
Single-cell restitution curves for epicardial (solid line, squares), midmyocardial (fine-dashed line, circles), endocardial (course-dashed line, triangles), and default (dot-dashed line, diamonds) parameter sets. Shown also at *left* of the plot is the maximum gradient of the restitution curve. APD, action potential duration.

Figure 4 shows single cell restitution plots for individual epicardial (solid line), midmyocardial (fine-dashed line), and endocardial (course-dashed line) cells with the corresponding adjustments to ionic currents described in *Electrophysiological heterogeneity assignment* in addition to the default cell (dot-dashed line). Immediately apparent in Fig. 4 is the respectively longer APD of the midmyocardial cells compared with endocardial cells and of endocardial cells compared with epicardial cells. This difference in APD is most apparent during high BCL but is significantly attenuated as pacing frequency increases. For example, at 500-ms BCL the APD of midmyocardial cells is 17.7-ms longer than that of the endocardial cells and 58.1-ms longer than the epicardial cells; at 180 ms, however, this is reduced to just 7.9 and 28.2 ms, respectively. Shown also in Fig. 4, *left*, are the maximum gradient values of the restitution plots. The endocardial cell has a maximum gradient close to that of the default cell (0.42 compared with 0.43), whereas the midmyocardial cell has a higher maximum gradient (0.64) and the epicardial cell a lower value (0.26).

#### Tissue level.

**Fig. 5.**
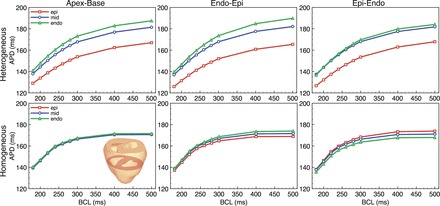
Comparison of restitution plots between models for different pacing protocols. Restitution plots of AP duration (APD) vs. BCL for heterogeneous (*top*) and homogeneous (*bottom*) models during apical (*left*), endo-epi (*middle*), and epi-endo (*right*) pacing. APDs for epicardial (red square), midmyocardial (blue circles), and endocardial (green triangles) shown represent mean APD values within each region through the mid-level slice shown in *bottom-left inset* (excluding septum).

Figure 5 shows restitution plots for the three different pacing protocols for both the heterogeneous and homogeneous models. APD values represent mean APDs for all nodes within each region (endocardial, midmyocardial, and epicardial) within the axial slice shown in Fig. 5, *bottom-left inset* (excluding septum). For all pacing directions, the magnitude of the difference in APDs between different transmural regions in the heterogeneous model is significantly attenuated, relative to the larger differences seen at the single cell level (due to electrotonic effects), as witnessed in Fig. 3. However, here we see that the smaller differences that are present at longer BCLs are attenuated even further as pacing frequency increases, similar to the effect seen in the single cell protocols above (Fig. 4). At a BCL of 180 ms, there is less than a 10-ms difference in APD between the different transmural regions for apex-base and epi-endo pacing directions and less than a 14-ms difference for endo-epi pacing.

**Fig. 6.**
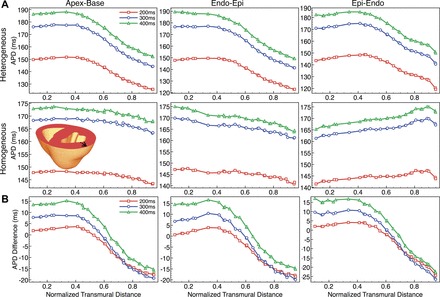
Transmural variation in APD for different pacing protocols. *A*: plots of APD vs. normalized transmural distance from endocardium along the transmural line through left ventricular free wall (shown in *bottom-left inset*) for heterogeneous (*top*) and homogeneous (*bottom*) models during apical (*left*), endo-epi (*middle*), and epi-endo (*right*) pacing. BCLs of 400 ms (green triangles), 300 ms (blue circles), and 200 ms (red squares) are shown. *B*: plots showing intrinsic difference in APD (heterogeneous model APD minus homogeneous) across the same transmural line as above for each pacing frequency.

We now examine how APD varies transmurally across the ventricular wall for different pacing directions. Figure 6*A* plots APD as a function of normalized transmural distance (along the transmural line shown at *bottom-left inset*) at 400-, 300-, and 200-ms BCLs for both heterogeneous and homogeneous models during all three pacing directions. In the homogeneous case during epi-endo and endo-epi propagation, we see the characteristic lengthening of the AP close to the stimulus site and shortening of the AP near the site of collision of the wavefront with the distal surface. During apex-base propagation, APD is largely homogeneous throughout the wall, with a slight shortening close to the epicardium due to the curved nature of the wavefront itself (Fig. 2) quickening repolarization in this region due to wavefront curvature (18). In the heterogeneous model, for all pacing directions the previously witnessed transmural dispersion of APD (Fig. 3*B*) is more visible, with a clear prolongation of APD from epi- to endocardium. However, the above-mentioned edge effects (increase in APD close to stimulus site, reduction in APD close to collision surface) combine with the intrinsic APD differences induced by the imposed transmural electrophysiological heterogeneity, accentuating the transmural dispersion of APD for endo-epi propagation and attenuating dispersion for epi-endo propagation. For example, total transmural dispersion in APD at a BCL of 300 ms is 35.2 ms for endo-epi propagation compared with 29.3 ms for epi-endo propagation. Figure 6*B* highlights the intrinsic heterogeneity remaining in the absence of edge-related electrotonic effects, showing the difference in APD between corresponding points in the two models (heterogeneous APD minus homogeneous APD). As can be seen in the difference plots, the remaining intrinsic heterogeneity in APD is similar for all pacing directions following the removal of electrotonic effects relating to pacing/wavefront direction.

Examination of APD variation along a line in the global apex-base direction also shows similar effects to those seen above (data not shown). In the homogeneous model case, APDs are very similar along the apex-base line during endo-epi and epi-endo pacing directions. For example, the difference in manifest APD between two points located at the apex and base during endo-epi pacing is approximately <2 ms at 200-, 300-, and 400-ms BCLs. During apical pacing, however, a slight reduction in APD (of ∼10 ms) is evident close to the base due to the electrotonic effects of wavefront collision with the cut surface. In the heterogeneous case, the enforced gradient of *I*_Ks_ conductance along the apicobasal direction leads to a noticeable intrinsic gradient in APD in this direction, as expected, with longer APDs close to the apex and shorter APDs near the base. For example, during endo-epi pacing, the difference in APDs between apex and base is >20 ms at each BCL, reaching ∼30 ms for apical pacing when electrotonic effects are additionally present.

### Arrhythmia Induction Via Pacing

#### Induction via rapid pacing.

**Fig. 7.**
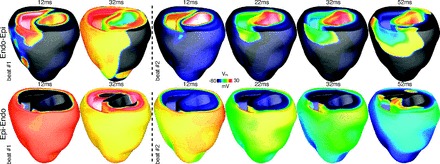
Arrhythmia induction following rapid endocardial (*top*) and epicardial (*bottom*) pacing. Images show *V*_m_ distributions within the ventricular model following successive pacing stimuli at a BCL of 170 ms. *Top* and *bottom*: successful planar conduction during *beat #1* and unsuccessful nonuniform conduction resulting in arrhythmia induction during *beat #2*.

Following the restitution protocols applied above on to the homogeneous and heterogeneous ventricular models, BCL was continually reduced in increments of 10 ms to attempt to induce an arrhythmia via rapid pacing. Arrhythmia induction was not possible in either model during apical pacing; below 180-ms BCL, conduction block and entire loss of capture resulted. However, during both endo-epi and epi-endo pacing arrhythmia induction was possible in the heterogeneous model. Figure 7 shows the mechanism of induction for both endo-epi (*top*) and epi-endo (*bottom*) pacing, respectively. Each panel shows *V*_m_ distributions at time instances between 12 and 42 ms following successive paced beats at BCL of 170 ms. In both cases, the first beat shown is successful, inducing 1:1 conduction and uniform wavefront propagation away from the pacing sites within the model. The second paced beat occurs before the tissue has fully recovered, inducing conduction block within most of the tissue surrounding the pacing sites. However, conduction is not blocked uniformly throughout the tissue and propagation eventually succeeds close to the insertion sites of the septum with the free wall (where heterogeneity is high). The heterogeneous distribution of refractory tissue following the largely failed paced beat then provides the essential substrate for these small wavefronts to interact with and generate sustained reentry. In the homogeneous model, arrhythmia induction was not possible during any rapid pacing protocol due to uniform conduction block and entire lack of capture as pacing frequency increased.

#### Induction via premature stimuli.

From the previous restitution analysis, it was shown that assigned intrinsic heterogeneity is retained to a greater extent during slower BCLs. Therefore, the application of a premature stimulus occurring during a slow pacing rate may exploit this heterogeneity more effectively to facilitate arrhythmia induction via a similar mechanism to that seen above in Fig. 7. To assess this, a premature stimulus was delivered to the ventricles via the pacing electrodes at different S2 CIs following prepacing at a BCL of 400 ms, as described in methods.

**Fig. 8.**
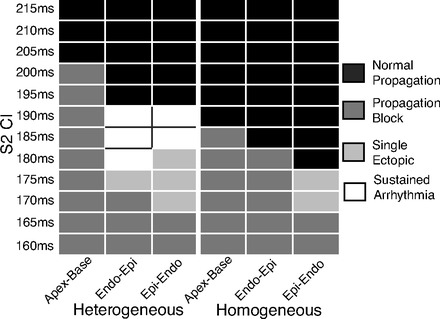
Vulnerability table for arrhythmia induction following premature stimulus for heterogeneous (*left*) and homogeneous (*right*) models during apex-base, endo-epi, and epi-endo pacing protocols. Colors represent protocol outcome following S2 stimulus: black, successful (normal) propagation; dark grey, complete conduction block and propagation failure; light grey, induction of a single, nonsustained ectopic beat; white, reentrant arrhythmia induction.

Figure 8 shows the vulnerability of each model for arrhythmia induction via this method for different CIs during different pacing electrode configurations. No arrhythmias were induced at any S2 CI via this protocol within the homogeneous model. Furthermore, neither model induced arrhythmic activity during apex-base pacing (only normal propagation or uniform block were observed). However, the heterogeneous model successfully induced arrhythmias during a number of CIs during epi-endo and endo-epi pacing, occurring between 180 and 190 ms. Arrhythmias were induced via similar mechanisms to those depicted in Fig. 7, involving nonuniform conduction block occurring in a number of regions. In addition, single ectopic beats were induced in the heterogeneous model, occurring when propagation was uniformly blocked except for one small region close to the base of the thin RV wall that produced a focal-like activation. After a short delay, propagation from this site simply swept through the rest of the recovered ventricles, resulting in a nonreentrant, ectopic beat. Such an ectopic activity was also seen in one case during epi-endo pacing in the homogeneous model.

### Sustained Arrhythmia Complexity

**Fig. 9.**
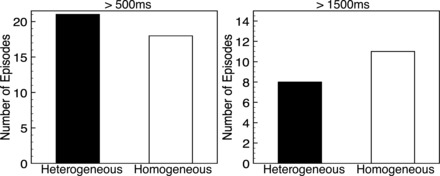
Number of induced arrhythmia episodes successfully sustained for longer than 500 ms (*left*) and 1,500 ms (*right*) for both heterogeneous (black) and homogeneous (grey) models.

To assess the role played by electrophysiological heterogeneity in sustained arrhythmias, a total of 36 different arrhythmias were induced following the protocol described in *Arrhythmia induction* and their resulting dynamics analyzed using the procedures outlined in *Data Analysis*. In each of the two models, similar numbers of episodes were initially successfully sustained (i.e., induced for >500 ms): 21 in the heterogeneous vs. 18 in the homogeneous model. Furthermore, similar numbers of episodes were subsequently self-sustained for a long duration (>1,500 ms): 8 in the heterogeneous vs. 11 in the homogeneous model, as demonstrated in Fig. 9.

**Fig. 10.**
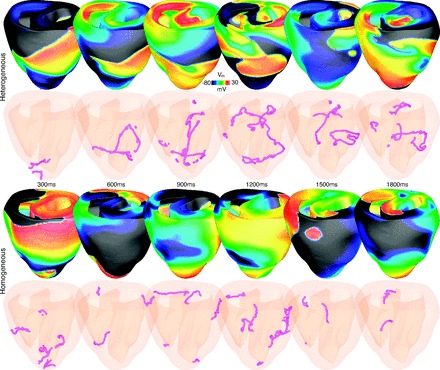
Evolution of arrhythmia dynamics. Images show *V*_m_ distributions and filaments (pink) within both the heterogeneous (*top*) and homogeneous (*bottom*) ventricular model during a sustained arrhythmia episode.

Visually, there was no discernible difference between the wavefront patterns or filament dynamics within in the heterogeneous vs. homogeneous models. This is demonstrated in Fig. 10, which shows *V*_m_ distributions (*top*) and filaments (*bottom*) at different time instances throughout the episodes for the two models. Both episodes were characterized by large wavefronts, sweeping around the ventricles. Wavebreak occurred relatively infrequently in each model and was not the dominant mechanism sustaining the arrhythmia.

**Fig. 11.**
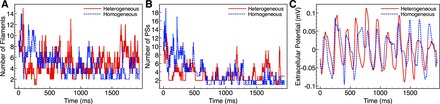
Evolution of filament (*A*) and phase singularities (PSs; *B*) counts during an arrhythmia episode (confidence interval = 190 ms; shock strengths = 25 V) for the heterogeneous (red solid line) and homogeneous (blue dashed line) models with the corresponding ECG (lead III) traces shown in *C*.

During sustained episodes, metrics quantifying the numbers of filaments and PSs and their respective dynamics were also similar between models. These similarities are demonstrated in Fig. 11, which shows, as an example, plots of the evolution in the number of filaments (*A*) and PSs (*B*) during two particular arrhythmias in the heterogeneous (solid line) and homogeneous (dashed line) models. For these episodes, the mean filament count was 4.10 in the heterogeneous model with a maximum at any time frame of 14, compared with 5.33 in the homogeneous model with a maximum of 15. In terms of PSs, the mean count was 4.06 in the heterogeneous model (maximum 14), compared with 4.41 in the homogeneous model (maximum 12).

Table 2 then summarizes the analysis shown as mean values averaged over all episodes sustained >1,000 ms in each model. The homogeneous model had arrhythmia episodes with slightly higher mean filament and PS counts, perhaps suggesting slightly more complex arrhythmia dynamics within the homogeneous model. However, other metrics such as maximum filament counts and total filament interaction rates were more similar between models. Furthermore, the heterogeneous model predicted shorter filament life times and longer mean filament lengths than the homogeneous model, suggesting potentially less stable dynamics in the heterogeneous model.

**Table 2.** Summary of filament dynamics metrics

Metric	Heterogeneous	Homogeneous
Filament nos.	3.71 ± 0.88	5.51 ± 1.08
Max filament nos.	14.10 ± 2.42	14.73 ± 2.25
PS nos.	3.37 ± 0.54	4.40 ± 0.77
Max PS	13.20 ± 1.62	16.27 ± 2.76
Mean filament length	327 ± 81	208 ± 29
Total interaction rate	0.64	0.71
Mean life times	11.36 ± 0.87	15.25 ± 1.28

Data are mean values ± SD obtained for all episodes sustained >1,000 ms. PS, phase singularities.

DF of the ECG is a commonly used metric to quantify the overall global complexity of an arrhythmic event. Table 3 shows that the mean DFs for each of the three ECG leads analyzed were very similar between the two models, which was also shown by the qualitative similarity in the ECG trace morphologies, an example of which is shown in Fig. 11*C*.

**Table 3.** Mean DFs obtained from ECG leads I, II, and III

Metric	Heterogeneous	Homogeneous
DF lead I	5.60 ± 0.72	5.44 ± 0.88
DF lead II	5.75 ± 0.32	5.62 ± 0.59
DF lead III	5.77 ± 0.50	5.44 ± 0.55

Data are mean dominant frequencies (DFs) ± SD obtained from ECG leads I, II, and III for all episodes sustained >1,000 ms in each model.

## DISCUSSION

Direct evidence of significant manifest electrophysiological heterogeneity in APD recordings from healthy intact hearts requires further elucidation (44). In tissue-level measurements where heterogeneity has been seen, it is always significantly attenuated compared with that witnessed in isolated cell recordings and, importantly, is almost entirely abolished at fast pacing rates. Consequently, the functional presence and role of APD gradients in the healthy ventricles, and particularly how heterogeneity may implicate itself in the dynamics of reentrant arrhythmias where tissue activation is rapid, remain incompletely understood, hampering the faithful construction of computational ventricular models used in arrhythmia investigations. By incorporating both intrinsic transmural and apicobasal heterogeneities in APD within a biventricular rabbit computational model, we have demonstrated that intrinsic APD gradients are significantly attenuated by tissue level electrotonic interactions and rapid pacing rates under a variety of different pacing protocols. Furthermore, electrotonic effects relating to activation direction were seen to further accentuate or attenuate the small manifest APD gradients that do remain, dependant on wavefront direction. Although the small degree of manifest APD heterogeneity at the fastest cycle lengths was sufficient to provide a substrate for arrhythmia induction (under the specific stimulation protocols analyzed), it was more relevant during premature stimuli application at slower pacing rates where intrinsic heterogeneity was less attenuated. Importantly, however, the dynamics of sustained reentrant arrhythmias within the whole ventricular model including physiological intrinsic gradients in assigned APD were in fact found to be very similar to those within an equivalent homogeneous model comparing a variety of different quantitative metrics.

### Electrotonic Modulation of Intrinsic APD Gradient During Pacing

The maximum magnitude of the transmural dispersion of APD at the tissue level found here was ∼35–45 ms (Fig. 5) at the slower pacing rates studied. This agrees well with previous experimental recordings from rabbit LV wedge preparations that reported an approximately 40-to 50-ms dispersion at stimulus rates of 1,000–2,000 ms. Furthermore, experimental measurements from the epicardial surface of isolated Landendorf-perfused rabbit hearts have also suggested apex-base APD dispersions of approximately 20–30 ms (4), which are again inline with those reported at the tissue level here. To our knowledge, however, the rate dependence of these variation has not been measured in the intact rabbit heart, limiting experimental validation. The transmural dispersion of APD predicted in our simulations was also inline with previous simulation studies that implemented similar experimentally derived transmural electrophysiological heterogeneity in ionic properties. Although these studies did predict slightly lower dispersions of APD (∼25 ms), this is thought to be due to their use of Luo-Rudy-based guinea pig, as opposed to rabbit, ventricular cell models. The preservation of a slightly higher transmural gradient of APD in our model therefore suggests that the results presented in this study represent somewhat of an upper limit of the role of heterogeneity on rapid pacing and arrhythmias in healthy rabbit ventricles.

The modulation of the intrinsic APD heterogeneity due to electrotonic interactions at the tissue level uncovered here, and its further reduction during higher pacing frequencies, agree with the findings from previous simulation studies on more anatomically simplistic models (15, 20, 21, 42). Electrotonic effects are known to be highly sensitive to specific wavefront shapes and myocardial fiber orientations (18). Here we importantly showed that such electrotonic modulation of intrinsic APD gradients exists within an anatomically realistic biventricular geometry incorporating realistic fiber architecture under a variety of different propagation directions. We believe that these findings, combined with the further modulation of heterogeneity at higher pacing frequencies, are therefore important in understanding the role intrinsic electrophysiological heterogeneity may play in arrhythmia mechanisms within the whole ventricles.

In spite of these electrotonic interactions, intrinsic APD heterogeneity was still noticeable, even under the fastest pacing cycles. However, recent transmural optical mapping studies from rabbit ventricular wedge preparations (38) have suggested that electrotonic influences alone may be sufficient to entirely overcome intrinsic differences in APD during transmural propagation, such that APD decreases from the pacing site for both epi-endo and endo-epi propagation. Such a relationship between activation time and APD was indeed present in our homogeneous model (Fig. 6), with APD longest close to the stimulation site and decreasing progressively away from it. In the heterogeneous case, this electrotonic effect interacted with the intrinsic transmural APD gradient, accentuating the overall dispersion of APD during endo-epi propagation and modulating it during epi-endo propagation. However, during epicardial stimulation, the decrease in manifest APD from endo to epi due to intrinsic APD heterogeneity was still present (Fig. 6). This apparent discrepancy between simulation and experiment could lie in previously discussed methodological concerns regarding the nature of these in vitro measurements (3, 50). Alternatively, it is possible that the appreciable wavefront curvature due to the point stimulus used in the experiments would increase electrotonic loading close to the stimulus site, further increasing APD preferentially in this region of high curvature, relative to the more planar wavefronts imposed from our simulation protocols (18, 21). The apparent reduced electrotonic interaction seen here could suggest that the assigned intrinsic heterogeneity in ionic currents is too severe and/or that diffusive coupling in the model is not sufficiently strong. However, assigned heterogeneity was based on experimental data and reproduced APDs within the experimental range for isolated cells and the experimentally derived conductivities (16) produced physiological conduction velocities. Indeed, the study by Clayton and Holden (15) was only able to completely overcome the intrinsic APD gradient in a simplified slab model through the use of conductivities that gave highly unphysiological conduction velocities.

### Importance of Intrinsic Heterogeneity During Sustained Arrhythmias

The main finding of this study is that inclusion of intrinsic electrophysiological heterogeneity within a computational model of the healthy rabbit ventricles makes little difference to the dynamics of induced arrhythmias. This supports previous computational modeling studies (5, 13, 47), which have used electrophysiologically homogeneous models, usually identifying this lack of heterogeneity as a limitation. The metrics used to assess arrhythmia dynamics and complexity within these studies, such as filament/PS counts, dominant frequencies, epicardial wavefront counts, as well as more qualitative descriptions of wavefront dynamics during reentrant behavior, have shown close agreement with experimental and clinical studies. Here, we show a direct comparison between simulated arrhythmias in electrophysiologically heterogeneous and homogeneous models of anatomically realistic healthy ventricles. The DFs recorded during the simulated arrhythmias (Table 3) corresponded to activation rates of ≈180 ms. At such rapid rates, intrinsic heterogeneity was significantly modulated under all wavefront propagation directions considered. This significant reduction in manifest APD gradients leads to very similar wavefront dynamics witnessed in the homogeneous model during episodes of reentry. These findings, therefore, underscore the suggestion that intrinsic APD gradients have little role in the dynamics of sustained arrhythmias in the healthy rabbit ventricles.

Despite overall similarities between the heterogeneous and homogeneous models during arrhythmias, small differences were still apparent. Interestingly, though, such differences were not as significant as suggested by studies performed on regular slab models (14, 15). These studies on less anatomically realistic geometries have shown that transmural APD heterogeneity can force filaments to move and stretch more, destabilizing both the filaments themselves and the reentry. We did find that the mean length of filaments in the heterogeneous case was longer than in the homogeneous model (also visually apparent to an extent in Fig. 10), perhaps indicative of stretching caused by the small remaining manifest APD heterogeneity at such fast activation rates. This increased filament length in the heterogeneous model may explain why, on average, slightly fewer filaments were seen relative to the homogeneous model (Table 2), as the longer filament lengths would reduce the number of reentrant circuits and scroll waves that could fit within the confines of the rabbit ventricular geometry (5, 31, 39). This is further reinforced by the fact that the total filament length (mean number multiplied by mean length) was very similar between models, suggesting the rabbit ventricles had reached the upper limit on the total filament length it could support. As there were slightly fewer mean filaments present in the heterogeneous model, the mean total interaction rate considered on a per filament basis, was slightly higher in the heterogeneous (0.17/filament) than the homogeneous (0.12/filament) model. This suggests that those filaments present in the heterogeneous model are slightly more unstable and interact more (both with other filaments and in terms of terminating and being created) than in the homogeneous model in line with the predictions of Clayton and Holden (14, 15). This is also supported by the slightly lower mean filament lifetime in the heterogeneous vs. homogeneous model.

Although minor differences were identified, the most important metrics describing the overall stability, dynamics, durations, and visual complexity of the arrhythmias were similar irrespective of electrophysiological heterogeneity. In contrast to the simplified geometries, the complex biventricular geometry incorporating realistic fiber architecture acts to dominate reentry dynamics, which we believe is driving the filament breakup in both models, despite the relatively shallow restitution curves. Consequently, the effects of electrophysiological heterogeneity are thus less apparent. Furthermore, simpler geometries have relatively more inexcitable boundaries, which encourage filament breakup, potentially increasing the apparent complexity relative to that predicted by ventricular models. In addition, the slab setups used previously had much thicker transmural walls (up to 12 mm; Refs. 14, 15) than in our rabbit geometry (LV, ∼6 mm; and RV, ∼3 mm), which is known to better preserve larger transmural APD gradients (42).

### Arrhythmia Induction Via Pacing

In this study, our focus was not to examine the role of electrophysiological heterogeneity during shock-induced arrhythmogenesis in which it has been previously demonstrated that intrinsic heterogeneity interacts with the applied extracellular stimulus in a variety of mechanisms specific to shock induction (33). However, our findings did successfully demonstrate the important role of intrinsic heterogeneity in the success of arrhythmia induction following both rapid pacing and premature paced beats.

During rapid pacing, we found that for epi- and endocardial stimulation, it was possible to induce reentrant activity within the heterogeneous model as pacing frequency increased, in contrast to the homogeneous model in which arrhythmia initiation was not possible for any pacing site. Although these findings could be specific to the exact protocols and electrode configurations used here, it appears that the small differences in manifest APD gradients that remain at the fastest cycle lengths, particularly in the apicobasal direction, become important in the formation of heterogeneous conduction block from the stimulus surface. We believe that entire epicardial and endocardial surface stimulation accentuates the interaction of the wavefronts with the intrinsically assigned apicobasal heterogeneity, which appeared to be modulated under fast pacing to a lesser degree than transmural gradients. It is important to note, though, that stimulation of smaller, perhaps more physiologically realistic, sites on the epi- and endocardium may not exploit this apex-base heterogeneity in this manner and thus arrhythmia induction may be harder. Indeed, the initial successful conduction pathway during both induction via epi- and enodocardial surface stimulation appeared close to the base where intrinsic APD is lower (Fig. 7). Although this proved an important mechanism during arrhythmia initiation, it did not appear to affect the overall dynamics of sustained arrhythmias (in which activation rate is also rapid), as discussed above. Such wholly transmural wavefront propagation patterns, perpendicular to the apicobasal APD gradient, would not be expected to occur frequently during arrhythmias due to the natural excitation pathways present in the ventricle. However, this mechanism could prove more important during the rapid firing of sinus Purkinje activity, which would induce wavefronts of this nature, although the longer APD of Purkinje cells may prevent such rapid excitation rates being achieved.

During the premature stimulus protocol, premature paced beats were applied to the ventricles during relatively slower paced rhythms, in which manifest APD heterogeneity was present to a greater degree than during faster BCLs. Such a scenario more faithfully replicates a potential mechanism of arrhythmia induction in vivo in which a premature beat may arise during normal sinus rhythm, for example, as a result of triggered activity or abnormal automaticity. Although this is potentially applicable to the case of endocardial stimulation (which approximately represents Purkinje activation), it should be noted that the stimulation electrodes used in this study to represent epicardial and apical stimulation do not represent physiologically realistic activation sites or sequences. Nonetheless, the more frequent occurrence of both arrhythmias and single ectopics seen in the heterogeneous model using these stimulus setups suggests that the presence of intrinsic heterogeneity is important for arrhythmia induction in the context of premature beats during slower rhythms where the remaining manifest heterogeneity is exploited.

Previous simulation (41) and experimental (28) studies have demonstrated the importance of APD gradients along the direction of pacing in the formation of unidirectional conduction block during premature stimuli, providing an arrhythmogenic substrate. These studies have shown that a point S2 stimulus (replicating focal triggered activity) applied to the wave-back can form unidirectional block in the presence of an APD gradient of ≥3 ms/mm. Although our choice of electrode configurations (at the extremities of the tissue) prevented a direct comparison with these previous works, it did allow us (for epicardial/endocardial stimuli) to exploit APD gradients in directions both parallel and perpendicular to the direction of pacing and importantly consider how they interact within the full three-dimensional anatomy of the ventricles. We were thus able to demonstrate how even relatively small apicobasal APD gradients may interact synergistically with transmural gradients and irregular ventricular wall thicknesses to allow nonuniform capture of the stimulus to induce reentry, a feature that was absent in the homogeneous model. Although less physiologically realistic, we believe that the protocols used here nonetheless uncover important insight relating to the combined role of transmural and apicobasal electrophysiological heterogeneities at the whole ventricle level in the mechanisms of reentry induction by a premature stimulus. These findings may be applicable to other, more physiological, scenarios, as well as under diseased conditions where electrophysiological heterogeneity (of a different type) may be more significant.

### Study Limitations

In this study we did not include a representation of the reduced electrical conduction in the subepicardial region, as has been suggested in some canine studies. Such an electrical uncoupling would be expected to reduce transmural electrotonic currents in the model. Given that here we only witnessed a modulation of intrinsic APD gradients by electrotonic effects, as opposed to the complete abolition demonstrated in previous experimental measurements (38), this would suggest that electrotonic coupling is, if anything, stronger, not weaker, than represented in our model, questioning the presence of such subepicardial uncoupling in the rabbit. Furthermore, although a recent study has suggested that M-cell populations are found in isolated islands within midmyocardial locations in the human (22), their exact distribution in the rabbit is still not known. Thus here our goal was to represent overall electrotonic effects of the presence of a large number of such specialized cells within the midmyocardium i.e., as a layer with increased APD, as has been done previously (14, 15, 33, 43).

Furthermore, our current model did not account for any transmural variation in intracellular calcium handling and the consequent heterogeneity in calcium transient that have been reported experimentally (27, 30). Including such variation could influence the transmural heterogeneity in AP morphology and duration through the bidirectional coupling between voltage and calcium within the cell. Furthermore, transmural variation in the calcium transient could also affect voltage via excitation-contraction coupling and mechano-electric feedback effects, which could also lead to important arrhythmogenic implications.

Finally, we would like to emphasize that in this study our focus is on the importance of healthy electrophysiological heterogeneity within physiological ventricles. We do not investigate the effects of heterogeneity due to remodeling or channelopathies in pathological ventricles, which is known to be significant (1, 46) and has been shown in previous modeling studies to increase the complexity of cardiac arrhythmias within a human ventricular model (25). We also highlight that the findings presented here are specific to the rabbit ventricles and similar enquires in other species will be necessary to draw overall conclusions related to the importance of intrinsic electrophysiological heterogeneity in sustaining arrhythmias. However, due to the known similarities in arrhythmia dynamics between the rabbit and the human (39, 45), we postulate that physiological heterogeneity may also play a lesser role in the human ventricles.

### Conclusions

We have shown that assigned intrinsic electrophysiological heterogeneity in APD in healthy ventricular cells is modulated by tissue-level electrotonic interactions and reduced further by rapid pacing rates under apex-base, epi-endo, and endo-epi propagation directions within an anatomically realistic ventricular model. As a consequence of this modulation, our findings further suggest that, although differences are witnessed during arrhythmia induction, the inclusion of electrophysiological heterogeneity does not significantly alter the dynamics of sustained reentrant arrhythmias in the healthy rabbit ventricle. This has important implications in the context of other previous studies, which have used homogeneous models in the investigation of arrhythmia mechanisms and in the future consideration of whether to represent such heterogeneity during construction of healthy ventricular models.

## GRANTS

M. J. Bishop is supported by the Wellcome Trust/EPSRC Medical Engineering Centre, King's College London. The study is supported by Austrian Science Fund FWF Grant F3210-N18 (to G. Plank) and National Heart, Lung, and Blood Institute Grant 1RO1-HL-10119601 (to G. Plank and E. J. Vigmond).

## DISCLOSURES

E. J. Vigmond and G. Plank are affiliated with Cardiosolv, LLC (Baltimore, MD).

## AUTHOR CONTRIBUTIONS

Author contributions: M.J.B., E.J.V., and G.P. conception and design of research; M.J.B. performed experiments; M.J.B. analyzed data; M.J.B., E.J.V., and G.P. interpreted results of experiments; M.J.B. prepared figures; M.J.B., E.J.V., and G.P. drafted manuscript; M.J.B., E.J.V., and G.P. edited and revised manuscript; M.J.B., E.J.V., and G.P. approved final version of manuscript.

## References

[1] Antzelevitch C Heterogeneity and cardiac arrhythmias: an overview. Heart Rhythm 4: 964–972, 2007 17599687 10.1016/j.hrthm.2007.03.036 PMC1950291

[2] Antzelevitch C Fish J Electrical heterogeneity within the ventricular wall. Basic Res Cardiol 96: 517–527, 2001 11770069 10.1007/s003950170002

[3] Anyukhovsky EP Sosunov EA Gainullin RZ Rosen MR The controversial M cell. J Cardiovasc Electrophysiol 10: 244–260, 1999 10090229 10.1111/j.1540-8167.1999.tb00667.x

[4] Banville I Gray RA Effect of action potential duration and conduction velocity restitution and their spatial dispersion on alternans and the stability of arrhythmias. J Cardiovasc Electrophysiol 13: 1141–1149, 2002 12475106 10.1046/j.1540-8167.2002.01141.x

[5] Bishop M Plank G The role of fine-scale anatomical structure in the dynamics of reentry in computational models of the rabbit ventricles. J Physiol 590: 4515–4535, 2012 22753546 10.1113/jphysiol.2012.229062 PMC3467803

[6] Bishop M Plank G Burton R Schneider J Gavaghan D Grau V Kohl P Development of an anatomically detailed MRI-derived rabbit ventricular model and assessment of its impact on simulation of electrophysiological function. Am J Physiol Heart Circ Physiol 298: H699–H718, 2010 19933417 10.1152/ajpheart.00606.2009 PMC2822578

[7] Bishop MJ Plank G Representing cardiac bidomain bath-loading effects by an augmented monodomain approach: application to complex ventricular models. IEEE Trans Biomed Eng 58: 1066–1075, 2010 21292591 10.1109/TBME.2010.2096425 PMC3075562

[8] Bishop MJ Plank G Bidomain ECG simulations using an augmented monodomain model for the cardiac source. IEEE Trans Biomed Imag 58: 2297–2307, 2011 10.1109/TBME.2011.2148718 PMC3378475 21536529

[9] Bishop MJ Vigmond EJ Plank G Cardiac bidomain bath-loading effects during arrhythmias: interaction with anatomical heterogeneity. Biophys J 101: 2871–2881, 2011 22208185 10.1016/j.bpj.2011.10.052 PMC3244060

[10] Cheng J Kamiya K Liu W Tsuji Y Toyama J Kodama I Heterogeneous distribution of the two components of delayed rectifier K^+^ current: a potential mechanism of the proarrhythmic effects of methanesulfonanilideclass III agents. Cardiovasc Res 43: 135–147, 1999 10536698 10.1016/s0008-6363(99)00061-9

[11] Cheng Y Li L Nikolski V Wallick DW Efimov IR Shock-induced arrhythmogenesis is enhanced by 2,3-butanedione monoxime compared with cytochalasin D. Am J Physiol Heart Circ Physiol 286: H310–H318, 2004 12958029 10.1152/ajpheart.00092.2003

[12] Clayton R Holden A A method to quantify the dynamics and complexity of re-entry in computational models of ventricular fibrillation. Phys Med Biol 47: 225–238, 2002 11837614 10.1088/0031-9155/47/2/304

[13] Clayton RH Vortex filament dynamics in computational models of ventricular fibrillation in the heart. Chaos 18: 043127, 2008 19123637 10.1063/1.3043805

[14] Clayton RH Holden AV Effect of regional differences in cardiac cellular electrophysiology on the stability of ventricular arrhythmias: a computational study. Phys Med Biol 48: 95–111, 2003 12564503 10.1088/0031-9155/48/1/307

[15] Clayton RH Holden AV Propagation of normal beats and re-entry in a computational model of ventricular cardiac tissue with regional differences in action potential shape and duration. Prog Biophys Mol Biol 85: 473–499, 2004 15142758 10.1016/j.pbiomolbio.2003.12.002

[16] Clerc L Directional differences of impulse spread in trabecular muscle from mammalian heart. J Physiol 255: 335–346, 1976 1255523 10.1113/jphysiol.1976.sp011283 PMC1309251

[17] Deo M Boyle P Plank G Vigmond E Arrhythmogenic mechanisms of the Purkinje system during electric shocks: a modeling study. Heart Rhythm 6: 1782–1789, 2009 19959130 10.1016/j.hrthm.2009.08.023 PMC5381712

[18] Fast VG Kléber AG Role of wavefront curvature in propagation of cardiac impulse. Cardiovasc Res 33: 258–271, 1997 9074688 10.1016/s0008-6363(96)00216-7

[19] Fenton F Karma A Vortex dynamics in three-dimensional continuous myocardium with fiber rotation: filament instability and fibrillation. Chaos 8: 20–47, 1998 12779795 10.1063/1.166374

[20] Franzone PC Pavarino LF Scacchi S Taccardi B Modeling ventricular repolarization: effects of transmural and apex-to-base heterogeneities in action potential durations. Math Biosci 214: 140–152, 2008 18621065 10.1016/j.mbs.2008.06.006

[21] Franzone PC Pavarino LF Taccardi B Effects of transmural electrical heterogeneities and electrotonic interactions on the dispersion of cardiac repolarization and action potential duration: a simulation study. Math Biosci 204: 132–165, 2006 16904130 10.1016/j.mbs.2006.06.002

[22] Glukhov A Fedorov V Lou Q Ravikumar V Kalish P Schuessler R Moazami N Efimov I Transmural dispersion of repolarization in failing and nonfailing human ventricle. Circ Res 106: 981–991, 2010 20093630 10.1161/CIRCRESAHA.109.204891 PMC2842469

[23] Grandi E Pasqualini FS Bers DM A novel computational model of the human ventricular action potential and Ca transient. J Mol Cell Cardiol 48: 112–121, 2010 19835882 10.1016/j.yjmcc.2009.09.019 PMC2813400

[24] Henriquez CS Simulating the electrical behavior of cardiac tissue using the bidomain model. Crit Rev Biomed Eng 21: 1–77, 1993 8365198

[25] Keldermann RH ten Tusscher KH Nash MP Hren R Taggart P Panfilov AV Effect of heterogeneous APD restitution on VF organization in a model of the human ventricles. Am J Physiol Heart Circ Physiol 294: H764–H774, 2008 18055526 10.1152/ajpheart.00906.2007

[26] Keller DU Weiss DL Dossel O Seemann G Influence of I(ks) heterogeneities on the genesis of the T-wave: a computational evaluation. IEEE Trans Biomed Eng 59: 311–322, 2012 21926009 10.1109/TBME.2011.2168397

[27] Laurita KR Katra R Wible B Wan X Koo MH Transmural heterogeneity of calcium handling in canine. Circ Res 92: 668–675, 2003 12600876 10.1161/01.RES.0000062468.25308.27

[28] Laurita KR Rosenbaum DS Interdependence of modulated dispersion and tissue structure in the mechanism of unidirectional block. Circ Res 87: 922–928, 2000 11073889 10.1161/01.res.87.10.922

[29] Li W Ripplinger C Lou Q Efimov I Multiple monophasic shocks improve electrotherapy of ventricular tachycardia in a rabbit model of chronic infarction. Heart Rhythm 6: 1020–1027, 2009 19560090 10.1016/j.hrthm.2009.03.015 PMC2813769

[30] Lou Q Fedorov VV Glukhov AV Moazami N Fast VG Efimov IR Transmural heterogeneity and remodeling of ventricular excitation-contraction coupling in human heart failure. Circulation 123: 1881–1890, 2011 21502574 10.1161/CIRCULATIONAHA.110.989707 PMC3100201

[31] Lou Q Li W Efimov IR The role of dynamic instability and wavelength in arrhythmia maintenance as revealed by panoramic imaging with blebbistatin vs. 2,3-butanedione monoxime. Am J Physiol Heart Circ Physiol 302: H262–H269, 2012 22037192 10.1152/ajpheart.00711.2011 PMC3334231

[32] Mahajan A Shiferaw Y Sato D Baher A Olcese R Xie LH Yang MJ Chen PS Restrepo JG Karma A Garfinkel A Qu Z Weiss JN A rabbit ventricular action potential model replicating cardiac dynamics at rapid heart rates. Biophys J 94: 392–410, 2008 18160660 10.1529/biophysj.106.98160 PMC2157228

[33] Maharaj T Blake R Trayanova N Gavaghan D Rodriguez B The role of transmural ventricular heterogeneities in cardiac vulnerability to electric shocks. Prog Biophys Mol Biol 96: 321–338, 2008 17915299 10.1016/j.pbiomolbio.2007.07.017 PMC2821334

[34] Manoach M Netz H Erez M Weinstock M Ventricular self-defibrillation in mammals: age and drug dependence. Age Aging 9: 112–116, 1980 10.1093/ageing/9.2.112 6104916

[35] McIntosh MA Cobbe SM Kane KA Rankin AC Action potential prolongation and potassium currents in left-ventricular myocytes isolated from hypertrophied rabbit hearts. J Mol Cell Cardiol 30: 43–53, 1998 9500863 10.1006/jmcc.1997.0570

[36] McIntosh MA Cobbe SM Smith GL Heterogeneous changes in action potential and intracellular Ca^2+^ in left ventricular myocyte sub-types from rabbits with heart failure. Cardiovasc Res 45: 397–409, 2000 10728360 10.1016/s0008-6363(99)00360-0

[37] Morita S Zipes D Morita H Wu J Analysis of action potentials in the canine ventricular septum: no phenotypic expression of M cells. Cardiovasc Res 74: 96–103, 2007 17266946 10.1016/j.cardiores.2007.01.003

[38] Myles RC Bernus O Burton FL Cobbe SM Smith GL Effect of activation sequence on transmural patterns of repolarization and action potential duration in rabbit ventricular myocardium. Am J Physiol Heart Circ Physiol 299: H1812–H1822, 2010 20889843 10.1152/ajpheart.00518.2010 PMC3006295

[39] Panfilov AV Is heart size a factor in ventricular fibrillation? or how close are rabbit and human hearts? Heart Rhythm 3: 862–864, 2006 16818223 10.1016/j.hrthm.2005.12.022

[40] Plank G Liebmann M Weber dos Santos R. Vigmond EJ Haase G Algebraic multigrid preconditioner for the cardiac bidomain model. IEEE Trans Biomed Eng 54: 585–596, 2007 17405366 10.1109/TBME.2006.889181 PMC5428748

[41] Qu Z Garfinkel A Weiss JN Vulnerable window for conduction block in a one-dimensional cable of cardiac cells, 1: single extrasystoles. Biophys J 91: 793–804, 2006 16679367 10.1529/biophysj.106.080945 PMC1563756

[42] Sampson KJ Henriquez C Electrotonic influences on action potential duration dispersion in small hearts: a simulation study. Am J Physiol Heart Circ Physiol 289: H350–H360, 2005 15734889 10.1152/ajpheart.00507.2004

[43] Saucerman JJ Healy SN Belik ME Puglisi JL McCulloch AD Proarrhythmic consequences of a KCNQ1 AKAP-binding domain mutation: computational models of whole cells and heterogeneous tissue. Circ Res 95: 1216–1224, 2004 15528464 10.1161/01.RES.0000150055.06226.4e

[44] Taggart P Sutton P Opthof T Coronel R Kallis P Electrotonic cancellation of transmural electrical gradients in the left ventricle in man. Prog Biophys Mol Biol 82: 243–254, 2003 12732283 10.1016/s0079-6107(03)00025-7

[45] tenTusscher K Mourad A Nash MP Clayton RH Bradley CP Paterson DJ Hren R Hayward M Panfilov AV Taggart P Organization of ventricular fibrillation in the human heart: experiments and models. Exp Physiol 94: 553–562, 2009 19168541 10.1113/expphysiol.2008.044065

[46] Tomaselli GF Marban E Electrophysiological remodeling in hypertrophy and heart failure. Cardiovasc Res 42: 270–283, 1999 10533566 10.1016/s0008-6363(99)00017-6

[47] Tusscher KT Hren R Panfilov A Organization of ventricular fibrillation in the human heart. Circ Res 100: e87–101 2007 10.1161/CIRCRESAHA.107.150730 17540975

[48] Vigmond E Hughes M Plank G Leon L Computational tools for modeling electrical activity in cardiac tissue. J Electrocardiol 36: 69–74, 2003 14716595 10.1016/j.jelectrocard.2003.09.017

[49] Vigmond EJ Weber dos Santos R. Prassl AJ Deo M Plank G Solvers for the cardiac bidomain equations. Prog Biophys Mol Biol 96: 3–18, 2008 17900668 10.1016/j.pbiomolbio.2007.07.012 PMC2881536

[50] Vos MA Jungschleger JG Transmural repolarization gradients in vivo: the flukes and falls of the endocardium. Cardiovasc Res 50: 423–5, 2001 11376616 10.1016/s0008-6363(01)00271-1

[51] Xie F Qu Z Yang J Baher A Weiss J Garfinkel A A simulation study of the effects of cardiac anatomy in ventricular fibrillation. J Clin Invest 113: 686–693, 2004 14991066 10.1172/JCI17341 PMC351312

[52] Xu X Rials SJ Wu Y Salata JJ Liu T Bharucha DB Marinchak RA Kowey PR Left ventricular hypertrophy decreases slowly but not rapidly activating delayed rectifier potassium currents of epicardial and endocardial myocytes in rabbits. Circulation 103: 1585–90, 2001 11257089 10.1161/01.cir.103.11.1585

[53] Yan GX Shimizu W Antzelevitch C Characteristics and distribution of m cells in arterially perfused canine left ventricular wedge preparations. Circulation 98: 1921–1927, 1998 9799214 10.1161/01.cir.98.18.1921

